# The Preschool Activity, Technology, Health, Adiposity, Behaviour and Cognition (PATH-ABC) cohort study: rationale and design

**DOI:** 10.1186/s12887-017-0846-4

**Published:** 2017-04-04

**Authors:** Dylan P. Cliff, Jade McNeill, Stewart Vella, Steven J Howard, Megan A. Kelly, Douglas J. Angus, Ian M. Wright, Rute Santos, Marijka Batterham, Edward Melhuish, Anthony D. Okely, Marc de Rosnay

**Affiliations:** 1 Early Start Research Institute, School of Education, Faculty of Social Sciences, University of Wollongong, Northfields Ave, Wollongong, NSW, 2522 Australia; 2 Illawarra Health and Medical Research Institute, University of Wollongong, Wollongong, Australia; 3 Early Start Research Institute, School of Psychology, Faculty of Social Sciences, University of Wollongong, Wollongong, Australia; 4 Faculty of Science, Medicine and Health, University of Wollongong, Wollongong, Australia; 5 School of Psychology University of New South Wales, Kensington, Australia; 6 Research Centre in Physical Activity, Health and leisure, Faculty of Sport, University of Porto, Porto, Portugal; 7 Statistical Consulting Centre, School of Mathematics and Applied Statistics, Faculty of Engineering and Information Sciences, University of Wollongong, Wollongong, Australia; 8 Department of Education, University of Oxford, Oxford, UK; 9 Psychological Sciences, Birkbeck, University of London, London, UK

**Keywords:** Early childhood, Physical activity, Active play, Screen time, Electronic media, Executive function, Theory of mind, Emotion understanding, Psychosocial, Well-being, Self-regulation, Cardiovascular

## Abstract

**Background:**

Prevalence estimates internationally suggest that many preschool-aged children (3–5 years) are insufficiently physically active and engage in high levels of screen-based entertainment. Early childhood is the developmental period for which we know the least about the effects of physical activity on development and health. Likewise, rapid technological advancements in mobile electronic media have made screen-based forms of entertainment for young children ubiquitous, and research demonstrating the impacts on cognition, psychosocial well-being, and health has lagged behind the rate of adoption of these technologies. The purpose of the Preschool Activity, Technology, Health, Adiposity, Behaviour and Cognition (PATH-ABC) study is to investigate if physical activity and screen-based entertainment are independently associated with cognitive and psychosocial development, and health outcomes in young children, and if so, how much and which types of these behaviours might be most influential.

**Methods:**

The PATH-ABC study is a prospective cohort, aiming to recruit 430 3–5 year-old children. Children are recruited through and complete initial assessments at their Early Childhood Education and Care (ECEC) centre, and then 12-months later at their centre or school. Direct assessments are made of children’s habitual physical activity using accelerometry, cognitive (executive function) and language development (expressive vocabulary), psychosocial development (emotional understanding, Theory of Mind, empathy, and heart rate variability), adiposity (body mass index and waist circumference), and cardiovascular health (blood pressure and retinal micro- vasculature). Educators report on children’s psychological strengths and difficulties and self-regulation. Parents report on children’s habitual use of electronic media and other child, parent and household characteristics.

**Discussion:**

The PATH-ABC study aims to provide evidence to enhance understanding of how much and which types of physical activity and screen-based media influence development and health in preschool-aged children. This information would benefit parents, educators, health professionals and governments seeking to develop strategies and policies to give young children the best start in life by promoting healthy levels of physical activity and electronic media use.

## Background

Prevalence estimates internationally suggest that substantial proportions of preschool-aged children (3–5 years) are insufficiently physically active and engage in high levels of screen-based entertainment [1–5]. For example, one Australian study indicated that only 5 and 20% of 3- to 5-year-olds met national guidelines for physical activity and electronic screen-based entertainment, respectively [2]. Understanding the impact of these prevalence rates should be a high priority, however early childhood is the developmental period for which we know the least about the independent effects of physical activity and screen behaviours on development and health [6–9]. Rapid technological advancements in mobile electronic media have made screen-based forms of entertainment for young children ubiquitous, and research demonstrating the influence on cognition, socialisation, behaviour, and health has lagged behind the rate of adoption [10]. Evidence indicates that physical activity is likely to improve young children’s weight status, bone health and motor development [6], however evidence for other health outcomes such as cardiometabolic health is less clear. Likewise, although the early years are characterised by rapid and formative changes in cognition, socialisation, and behaviour [11], the independent influences of habitual physical activity and screen-based entertainment on cognitive and psychosocial development among preschool children are not well understood [7–9].

Emerging evidence suggests that physical activity may affect school-aged children’s higher-order cognitive processes, such as executive functions, that in turn affect self-regulation [12–14]. Self-regulation is a construct composed of multiple inter-related high-level cognitive skills responsible for formulating goals, planning how to achieve them, and carrying out these plans effectively [15]. These executive functions develop throughout childhood and include inhibition, cognitive flexibility, and working memory [15]. Self-regulation and executive functions are strong indicators of school readiness and later academic achievement [16], and are intimately linked to children’s psychosocial development and behaviour [17]. In a seminal experimental study, Davis and colleagues [12] found that regular physical activity improved executive functions and altered brain activity in inactive overweight 7- to 11-year-olds. Remarkably, children’s ability in mathematics improved despite the fact they received no direct instruction in this area. Nevertheless, the influence of physical activity on cognition and executive function in young children is not well established [8]. Consequently, it remains unclear if physical activity aids cognitive development in early childhood, and if so, how much physical activity, of what intensity, and which types might be required for optimal development.

Psychosocial development encompasses children’s emotional, behavioural and social functioning. In Australia, approximately 50% of 2–3 year-olds exhibit externalising (e.g., aggression) and internalising (e.g., anxiety) behavioural problems “sometimes” or more often, and 10% of 4–5 year-olds display consistent behavioural problems in these areas [18]. These behavioural problems can be persistent and can place children at risk of problematic developmental pathways [19]. A recent systematic review indicated that there are too few studies to draw conclusions about associations between electronic media use or physical activity and psychosocial development in young children [7]. Studies in this area have investigated a narrow range of screen-based behaviours, with only one of seven studies in 3–5 year-olds examining multiple screen behaviours beyond TV viewing [20]. Likewise, studies have typically not controlled for the potentially beneficial effects of physical activity, as only two studies concurrently assessed electronic media behaviours and physical activity [20, 21]. Of the studies that have examined associations between physical activity and psychosocial development, only one study used an objective measure of physical activity [21], which raises concerns about measurement accuracy, and all but one study [22] used a cross-sectional design, from which it is difficult to determine if physical activity is the cause or consequence of psychosocial outcomes. Furthermore, there is little research on relations between physical activity and media use, on the one hand, and children’s social cognition, on the other hand. Social cognition, which includes children’s empathy and understanding of mind and emotion, has been shown to be an important predictor of early social behaviour and integration [23–25] that is highly sensitive to the child’s social and communicative environment [26]. Physical activity, which often involves group based participation, and media use, which removes young children from interpersonal communications, might both exert indirect effects on children’s social behaviour (e.g., behaviour problems, social skills, prosocial behaviour, etc.) through their potential influences on the development of social cognitive capacities, which have been shown to change rapidly in the preschool period [27] and rely heavily on executive functions [28]. In keeping with this interpretation, there is limited cross-sectional research which suggests that high levels of television viewing may be detrimentally associated with young children’s social cognition [29]. Finally, to our knowledge, objective psychophysiological assessments of emotional and social development, such as heart-rate variability, have not been collected in population samples of young children with concurrent assessments of lifestyle behaviours such as electronic media use or physical activity. Understanding if physical activity or electronic media use are associated with psychophysiological biomarkers would assist in explaining potential pathways through which these lifestyle behaviours might influence young children’s psychosocial development.

Investigating associations of lifestyle behaviours with cardiovascular and diabetes-related health outcomes in young children is difficult, because invasive clinical measures such as blood tests are not appropriate for population samples. However, non-invasive measures such as *retinal micro-vasculature assessments* could provide important insights into the early development of cardiovascular structure. The blood vessels of the retina can be assessed non-invasively in the field using portable retinal imaging and this measure has been used as a marker of systemic micro-vasculature. In adults, changes in retinal vascular caliber (narrower retinal arteriolar caliber and wider venular caliber) predict higher risks of hypertension and diabetes [30], higher risk for stroke [31], coronary heart disease [31], and coronary heart disease mortality [32]. Retinal micro-vascular changes, at least in adults, appear to be structural markers of subclinical cardiovascular disease and predict future vascular events [33, 34]. Furthermore, in adulthood, retinal micro-vasculature assessments provide clinically meaningful information on risk of cardiovascular disease, beyond conventional risk factors such as blood pressure [31]. Notably, deleterious retinal vascular changes are likely to be present before the manifestation of other cardiovascular disease risk factors such as elevated blood pressure in young children [35]. Paediatric studies demonstrate that: i) higher blood pressure and body mass index (BMI) at 3–5 years of age [35], and ii) higher levels of television use and lower levels of participation in parent-reported outdoor sporting activity as early as 6 years of age [36], were associated with adverse retinal micro-vasculature development. However, the nature of the associations between objectively measured physical activity and electronic media use with retinal micro-vasculature in preschool-aged children remain unknown.

In recognition of the potential health and developmental benefits for young children of increasing physical activity and reducing screen-based entertainment, several countries including Australia [37], Canada [38], and the United Kingdom (UK) [39], have released guidelines for physical activity and sedentary behaviours. Consistent with the guidelines released in other countries, the Australian *National Physical Activity Recommendations for Children 0*–*5 years* specify that 3–5 year-olds should be physically active for ≥3 h/day, and that their screen-based entertainment should be limited to <1 h/day [37]. There is, however, ongoing discussion surrounding these recommendations [40] because the amount and intensity of physical activity [e.g., light- (LPA), moderate- (MPA), or vigorous-intensity physical activity (VPA)], and the amount and types of screen behaviours that might influence development and health in 3–5 year-olds remain unclear [6–9]. Scholars have highlighted the need for further evidence to understand the impact of physical activity and screen-based behaviours on development and health among young children [6–9, 40].

The aim of the *Preschool Activity, Technology, Health, Adiposity, Behaviour, and Cognition* (PATH-ABC) study is to contribute to addressing this knowledge deficit by investigating the following research questions among 3–5 year-old children, both cross-sectionally and longitudinally:

Are physical activity and use of screen-based entertainment independently associated with cognitive and psychosocial development and health outcomes?

- Do the associations differ by the intensity (e.g., LPA or moderate-to-vigorous-intensity physical activity (MVPA)), amount, or type of physical activity?
- Do the associations differ by the type (e.g., TV/DVDs or computer/portable electronic devices) and amount of screen-based entertainment or the total amount of sedentary behaviour?
- Do the associations differ by sex, age or socioeconomic position?

## Methods and design

### Study design

The PATH-ABC study is a prospective cohort study investigating independent associations of physical activity and electronic media use with developmental and health outcomes in young children.

### Setting, sampling and recruitment

Participating children were recruited from Early Childhood Education and Care (ECEC) centres in the Illawarra region of New South Wales, Australia between April and December 2015. Centres were invited to participate based on a stratified sampling process. Firstly, all ECEC centres in the region were categorised into low (deciles 1–4), medium (deciles 5–7) or high (deciles 8–10) socio-economic areas based on their suburb and the 2011 Socio-Economic Indices For Areas (SEIFA) Index of Relative Socio-economic Advantage and Disadvantage (IRSAD) [41]. The number of ECEC centres invited to participate from each socio-economic group was proportional to the number within the population of centres. Children were assessed at Time 1 (age 3–5 years; April to December 2015) and 12 months later at Time 2 (April to December 2016). Data collection occurred in children’s ECEC centres, including pre-schools and long-day care centres, or primary school at Time 2, by 2–4 trained assessors over 1–5 days.

### Participants

Children were eligible to participate in the study if they attended an ECEC centre and were ≥3 years-of-age, generally healthy, and experiencing typical development. Children were ineligible if they had a learning or physical disability, known motor delay, or a diagnosed medical or psychological condition (e.g., conduct disorder) that would affect the results of the study. The exclusion criteria are based on the need to first understand the associations between physical activity, screen-based behaviours, and developmental outcomes in typically developing preschoolers, prior to exploring associations in specific groups.

### Study protocol

Participants and their parents/carers followed the same measurement protocol at each time point. Prior to participation in the study, Directors of participating ECEC centres provided electronic or paper versions of information sheets and consent forms to all eligible children’s parents/carers. Following informed consent, parents/carers provided demographic information and other data via surveys. Educators completed surveys reporting on participating children’s self-regulation and their psychological strengths and difficulties. After verbal consent, trained data collectors completed assessments with children in a quiet area of the ECEC centre, away from the main group of children but within the supervision of the educators. Measures were completed in assessment sessions grouped by outcomes (cognitive development, social/emotional development, and physical health), with assessors being flexible and sensitive to children’s need to take breaks between assessments.

### Measures

#### Physical activity

Physical activity was measured objectively using accelerometers (ActiGraph GT3X+), which are the method of choice for monitoring habitual activity behaviour in young children. The ActiGraph has established acceptability, validity and reliability in preschool children [42, 43]. Procedures will be in line with best-practice recommendations for using activity monitoring in young children [43]. At each time point children wore an accelerometer around their waist on an elastic belt at the right hip, anterior to the iliac crest, 24 h per day for one week. As habitual sedentary time is a potential determinant of children’s development and health, independent of physical activity, total sedentary behaviour will also be captured. Sedentary behaviour, LPA, MPA, and VPA will be defined using age-appropriate cut-points that have been shown to be most accurate in young children [5, 42, 44]. Outcomes will also include activity counts per epoch as an indicator of children’s average activity level.

#### Screen-based entertainment

Parents reported on the time children usually spend in screen-based behaviours during a typical week using questionnaire items with established reliability [2]. This includes the total amount of time their child spent in separate screen-based behaviours during the week (Monday to Friday) and on weekends (Saturday and Sunday). Original items were modified to include mobile devices as follows: television programs/movies/internet clips on traditional devices (e.g., TV/DVD), television programs/movies/internet clips on other devices (e.g., tablet, DVD in car, computer, laptop, mobile phone), games/apps on portable handheld devices (e.g., tablet, mobile phone, handheld game system), console games (non-active) (e.g., PlayStation, Xbox), and console games (active) (e.g., Wii, Xbox Kinect). Weekday and weekend day minutes will be summed across the week to calculate total weekly time spent in each behaviour. Subsequently, weekly totals for each behaviour will be summed to derive the child’s total weekly time spent in screen-based entertainment.

#### Cognitive and language development

The Early Years Toolbox (EYT - http://www.eytoolbox.com.au) is a readily available battery of iPad-based executive function, language, self-regulation, and social development measures that have been designed and psychometrically tested with preschool-aged children [15]. Four tasks were used, to assess key executive function components; 1) visual-spatial working memory (‘Mr Ant’) and phonological working memory (‘Not This’) 2); inhibition (‘Go/No-Go’); and 3) task shifting (‘Card Sorting’). A fifth task assesses expressive vocabulary. All tasks are self-contained and are “game-like” assessments implemented using an electronic tablet. Each measure was designed to be brief (≤5 min, including instruction and practice), engaging, and leverage the affordances of technology (e.g., animation, audio, and accurate capture of responses and response timings). All instructional and practice trials provide feedback to participants. Each task provides standardised administration and instructions visually and aurally, with instructions supplemented by the data collector if required. This test battery provides both methodological and practical advances, with established validity, reliability and developmental sensitivity [15].

#### Psychosocial development

*Child psychological strengths and difficulties, and self-regulation.* The Strengths and Difficulties Questionnaire (SDQ) was used to assess children’s psychosocial development [45]. The SDQ includes 25 items assessing five subscales: conduct problems, hyperactivity*,* emotional symptoms*,* peer problems and pro-social behaviour*.* Each item (i.e., child behaviour) is rated in frequency on a 3-point Likert Scale, and has established validity and reliability in young Australian children [46]. Psychological difficulties will be calculated by summing the ‘problem-based’ subscales: conduct problems, hyperactivity, emotional symptoms*,* and peer problems. Sub-scales can be combined to examine internalising problems (the sum of emotional symptoms and peer relationship problems subscales)*,* externalising problems (the sum of conduct problems and hyperactivity subscales) and pro-social behaviours. In line with a previous extension of the SDQ (14), an additional 15 questions were added to this questionnaire specifically tapping into children’s self-regulation constructs: cognitive (e.g., can work easily with others), behavioural (e.g., likes to work things out for self; seeks help as last resort) and emotional self-regulation (e.g., shows wide mood swings) (total of 40 questions) [15]. Educators/teachers were asked to complete the questionnaire for all consenting children involved in the study at both time points.

*Emotional understanding and Theory of Mind (ToM).* To understand children’s social and emotional development, three test batteries including the Test of Emotion Comprehension [47], the Assessment of Children’ s Emotion Skills [48] and Theory of Mind [27], which have both been shown to be valid assessments of children’s social cognitive understanding, were administered.

Four components of the Test of Emotion Comprehension (TEC) test battery were administered to assess participants’ emotional understanding, namely: *recognition* (I), *external cause* (II), *desire* (III), and *belief* (IV; [47]. For the first TEC component, recognition, children are asked to point to emotional expressions that correspond to five different emotion labels (i.e., *happy*, *sad*, *angry*, *scared* and *just okay*). This first item establishes whether children understand the correspondence between conventional emotion labels and canonical, simplified expressions. In subsequent TEC components, children’s responses are given by pointing at a selection (2 × 2) of emotion expressions (e.g., happy, sad, angry, just alright) following a story scenario that has an emotional outcome or consequence for the protagonist. Thus children’s responses are closed and non-verbal. For example, in component II, external causes, children are shown a picture, read an accompanying story, and are asked to select an emotional outcome (presented as four emotion faces) that would be provoked by an external cause (e.g., a Birthday party) displayed in each of five separate vignettes. Details for components III and IV are provided in Pons et al. [47]. The TEC yields a *competence rating* for each component (pass/fail), or can be scored for each item (components I-IV have 23 items).

In order to assess understanding of more naturalistic emotion expressions, children were administered a task assessing their ability to match real emotional facial expressions (presented as photographs) with correct labels, based on the Assessment of Children’s Emotion Skills (photographs of school-aged children expressing different facial expressions; see [24] and [48]). Children were shown two sets of five emotion expressions (two sets: happy, sadness, anger, fear, and surprise) and were asked to point to each nominated expression (in a preordered, random sequence). Children did not have to produce labels; they merely had to indicate their best answer. One point was awarded for each correctly identified emotion. The total time for these emotion understanding assessments is approximately 10 min.

A scaled set of age-appropriate tasks from the global assessment used by Wellman and Liu [27] were used to assess Theory of Mind (ToM). These tasks are variants of widely used ToM tasks and validation of the battery has been demonstrated preschoolers and young children [27]. These tests assess how a child is able to attribute core mental states to others; specifically, desires, knowledge, true beliefs, false beliefs, and concealed emotions. The scaling ToM assessment has be widely used internationally and is the current bench mark by which ToM development is assessed in the preschool period [49]. Each task is administered in a specific order due to the logical progression of a child’s development. All tasks require the participants to answer one target question depicted in a story book about a protagonist’s mental state or behaviour and one control or contrast question about reality or someone else’s mental state or behaviour. These tasks can be completed in approximately 5 min per child and children are awarded a pass/fail response for each of the five items.

*Empathy.* Empathy, as defined here, can be differentiated from social cognition as it is the emotional response that stems from the comprehension of another person’s affective state, and results in feelings that are similar to that other person [50]. Empathy facilitates individuals’ socially competent interactions, motivates helping and the desire for justice for others, and inhibits aggression towards others [51]. Although it has been proposed that increased exposure to electronic media might displace young children’s interactions with caregivers and peers, subsequently impeding the development of social skills such as empathy [10], there is little evidence to substantiate this hypothesis [7]. A revised 7-item version of the Index of Empathy for Children and Adolescents was used to assess empathy [52]. The items are administered using a video portraying two puppets depicting opposing responses. For each item, one puppet represents a more empathetic response, while the other represents a less empathetic response; however the more empathic response is varied systematically across the two puppets. The child is then asked to point to the puppet whose response is most similar to how they would feel. E.g., Statement 1: More empathetic puppet: *I get upset when I see a girl being hurt*. Less empathetic puppet: *I don’t get upset when I see a girl being hurt.* Item scores are combined to provide a total score out of 7, with higher scores indicated a more empathic response pattern.

#### High Frequency Heart Rate Variability (HF-HRV)

Cardiac parasympathetic outflow is routinely estimated by calculating variation in the length of successive cardiac cycles associated with respiration, referred to as High Frequency Heart Rate Variability (HF-HRV). Parasympathetic outflow to the heart is primarily meditated by the vagus nerve, and this outflow is modulated by respiration. During inhalation, cycle lengths rapidly decrease, while during exhalation they rapidly increase. This cardiorespiratory phenomenon is referred to as respiratory sinus arrhythmia, and individual differences in resting respiratory sinus arrhythmia have been correlated with individual differences in cognitive and psychosocial performance [53, 54]. Because the duration of cardiac cycles fluctuates with respiration, estimates of respiratory sinus arrhythmia are simple to obtain, first using non-invasive electrocardiogram (ECG) recording devices, and then by quantifying the statistical variation in the duration of heart beats over time (e.g., root mean square of successive differences), or using methods that calculate the amplitude of variation in cardiac cycles corresponding to the respiratory frequency (0.15Hz - 0.40Hz).

Because of the ease of collection and quantification, HF-HRV is ideal for identifying parasympathetic mediated correlates of adaptive and maladaptive behaviour and psychosocial function. Although experimental evidence in overweight and obese 7–11 year-old children has demonstrated that increasing physical activity can alter HF-HRV [55], associations between physical activity or screen-based entertainment and HF-HRV have not, to our knowledge, been investigated in 3–5 year-old children.

In the PATH-ABC study, ECG was recorded using a non-invasive, lightweight (13 g) ambulatory monitor (eMotion Faros 180°, Mega Electronics Ltd), with the beginning and end of the data window identified using built in event marker functionality. Recording sites on the right and left collar bone and left hip were cleaned using disposable alcohol wipes, residual alcohol was removed using gauze, and ECG sensors were attached. HF-HRV data were quantified during two conditions. Firstly, baseline or resting data were collected while children sat quietly for 5 min watching an e-book on an electronic tablet (Guess How Much I Love You: https://www.youtube.com/watch?v=Ze-XaKq3gU0), listening through headphones to block-out background noise. An emotionally-mild e-book was chosen to maintain children’s attention and reduce the impact of artifacts or “noise” in the data. Pilot testing of other “baseline” methods such as asking children to sit still or to complete a “vanilla task” [56] indicated that these methods were inappropriate for the age of the current participants (e.g., excessive movement artifacts, early termination of recording). Secondly, HF-HRV data were collected while children completed one executive function task (Go-No-Go) for approximately 4–5 min, the challenge from which was expected to heighten children’s attention and anxiety, and decrease HF-HRV. These recording periods were chosen to ensure that there is sufficient artefact free data for accurate estimation of parasympathetic outflow.

#### Adiposity

Height was measured without shoes to the nearest 0.1 cm using a portable stadiometer (Seca 217). Body mass was measured without shoes and in light clothing to the nearest 0.1 kg using a digital scale. Body Mass Index (BMI – weight (cm)/height (m)^2^) will be calculated. Waist circumference was measured using a non-extensible steel tape at the level of the superior border of the iliac crest. All measures were conducted twice. If the two measures were more than 0.5 cm different for height or waist circumference, and more than 0.5 kg different for the child’s weight, a third measure was taken, and the closest two are averaged.

#### Retinal imaging

Digital retinal photographs centred on the optic disc were obtained for both eyes using a portable non-mydriatic fundus camera (Smartscope Pro, Optomed, Finland). A semi-automated computer imaging analysis program, Singapore “I” Vessel Assessment (SIVA) (Exploit Technologies Pte Ltd, Singapore) will be used to measure the caliber of all retinal vessels within 0.5–1.0 disc diameter from the optic disc, following a standardized protocol using the revised Knudtson–Parr–Hubbard formula [57, 58]. The average arteriolar and venular calibers, summarized as the central retinal arteriolar equivalent (CRAE) and the central retinal venular equivalent (CRVE), is calculated from the six largest arterioles and venules. The right-eye image will be assessed for each participant, where this image is ungradable, the left eye image will be measured. One assessor will perform all the retinal vascular caliber measurements and intra-assessor reliability will be assessed on a randomised sub-sample of images.

#### Blood pressure

Systolic and diastolic blood pressure was taken twice using a portable sphygmomanometer (Welch Allyn Connex ProBP 3400) with the appropriate cuff size around the upper left arm. The child was asked to sit in a relaxed position while watching an age-appropriate video, to reduce movement.

#### Additional measures

In addition to the above measures, data on potential predictors or covariates were collected by direct assessments or parent-reports. These include children’s age, sex, indigenous status, country of birth, birth weight and gestational age at birth, usual dietary intake (intake of core and convenience/junk foods and on foods that may be considered as convenience/junk foods), sleep duration, and participation in extra cost leisure and educational activities including sport participation and music lessons [59]. Information was also collected on primary care-giver demographics (age, highest level of education, marital and employment status), household characteristics (primary language spoken at home, home learning environment [60], household income), electronic media device ownership, and parental history of lifestyle-related health conditions.

### Data analysis

Cross-sectional and longitudinal associations will be tested using linear regression or generalized linear models, where appropriate. Models will include exposures (e.g., MVPA or TV) and covariates (e.g., socioeconomic position) as fixed factors, preschool (cluster) as a random factor, and developmental and health measures (e.g., executive function) as the outcome. Exposures will be examined as continuous and categorical outcomes (e.g., quartiles) to understand dose-response relationships. Longitudinal analyses will examine: i) Time 1 exposure predicting Time 2 outcome (adjusting for Time 1 outcome), and ii) change in exposure predicting change in outcome. Interactions will be used to test for differences in associations by sex, age, or socioeconomic position.

Limited evidence in older children suggests that moderate to weak associations exist between physical activity or screen-based entertainment and cognitive, psychosocial or physical health outcomes [6–9]. In order to detect weak associations (*R* ^*2*^ = 0.06, *r* = 0.25) as significant (*p* = 0.05), with 0.80 power, and with a maximum of eight predictors/covariates in a comprehensive model, 257 participants would need to have complete data at Time 2. Accounting for 15% missing data at each of the two time points and a design effect, to adjust for the clustered nature of the data, of ~1.2 (based on an average cluster size of ~20 and an ICC of ~0.01), we aimed to recruit 430 participants to the study.

## Discussion

The PATH-ABC study, to our knowledge, represents one of the first comprehensive prospective investigations to simultaneously examine associations of objectively-measured physical activity and screen-based entertainment with novel cognitive and psychosocial development and other health outcomes in preschool children. This project could meaningfully inform physical activity and screen behaviour guidelines for the early years internationally, because prospective studies with measures of screen behaviours and objective measures of physical activity such as the one proposed have been identified as critical to enhancing the quality of the evidence base [6–9, 40]. Findings from this study will provide a better understanding of how much and which intensities or types of physical activity and screen-based entertainment influence development and health in preschool children. This knowledge could potentially be beneficial, for: i) parents aiming to optimise developmental outcomes for their children; ii) early childhood educators, clinicians, health care providers, and health promoters, aiming to enhance developmental outcomes in young children through evidence-informed intervention programs; iii) government departments seeking to develop evidence-based guidelines; and iv) researchers seeking to develop, evaluate and translate effective programs to give young children the best start in life.

## Abbreviations

BMI: Body mass index
CRAE: Central retinal arteriolar equivalent
CRVE: Central retinal venular equivalent
DVD: Digital versatile disk
ECEC: Early childhood education and care
ECG: Electrocardiogram
IRSAD, HF-HRV: High frequency heart rate variability; index of relative socio-economic advantage and disadvantage
LPA: Light-intensity physical activity
MPA: Moderate-intensity physical activity
MVPA: Moderate-to-vigorous-intensity physical activity
PATH-ABC: Preschool Activity, Technology, Health, Adiposity, Behaviour and Cognition study
SDQ: Strengths and difficulties questionnaire
SEIFA: Socio-economic indices for areas
SIVA: Singapore “I” vessel assessment
TEC: Test of emotional comprehension
ToM: Theory of mind
TV: Television
VPA: Vigorous-intensity physical activity
YET: Early years toolbox

## References

[1] Beets MW, Bornstein D, Dowda M, Pate RR (2011). Compliance with national guidelines for physical activity in US preschoolers: measurement and interpretation. Pediatrics.

[2] Hinkley T, Salmon J, Okely AD, Crawford D, Hesketh K (2012). Preschoolers’ physical activity, screen time, and compliance with recommendations. Med Sci Sports Exerc.

[3] Colley RC, Garriguet D, Adamo KB, Carson V, Janssen I, Timmons BW, Tremblay MS (2013). Physical activity and sedentary behavior during the early years in Canada: a cross-sectional study. Int J Behav Nutr Phys Act.

[4] De Craemer M, Lateva M, Iotova V, De Decker E, Verloigne M, De Bourdeaudhuij I, Androutsos O, Socha P, Kulaga Z, Moreno L (2014). Differences in energy balance-related behaviours in European preschool children: the ToyBox-study. Sci Sport.

[5] Pate RR, O'Neill JR, Brown WH, Pfeiffer KA, Dowda M, Addy CL (2015). Prevalence of compliance with a new physical activity guideline for preschool-age children. Childhood Obesity.

[6] Timmons BW, LeBlanc AG, Carson V, Connor Gorber S, Dillman C, Janssen I, Kho ME, Spence JC, Stearns JA, Tremblay MS (2012). Systematic review of physical activity and health in the early years (aged 0–4 years). Appl Physiol Nutr Metab.

[7] Hinkley T, Teychenne M, Downing KL, Ball K, Salmon J, Hesketh KD (2014). Early childhood physical activity, sedentary behaviors and psychosocial well-being: a systematic review. Prev Med.

[8] Carson V, Hunter S, Kuzik N, Wiebe SA, Spence JC, Friedman A, Tremblay MS, Slater L, Hinkley T (2016). Systematic review of physical activity and cognitive development in early childhood. J Sci Med Sport.

[9] LeBlanc AG, Spence JC, Carson V, Connor Gorber S, Dillman C, Janssen I, Kho ME, Stearns JA, Timmons BW, Tremblay MS (2012). Systematic review of sedentary behaviour and health indicators in the early years (aged 0–4 years). Appl Physiol Nutr Metab.

[10] Radesky JS, Schumacher J, Zuckerman B (2015). Mobile and interactive media use by young children: the good, the bad, and the unknown. Pediatrics.

[11] Shonkoff JP, Philips D (2000). From neurons to neighborhoods: the science of child development.

[12] Davis CL, Tomporowski PD, McDowell JE, Austin BP, Miller PH, Yanasak NE, Allison JD, Naglieri JA (2011). Exercise improves executive function and achievement and alters brain activation in overweight children: a randomized, controlled trial. Health Psychol.

[13] Diamond A, Lee K (2011). Interventions shown to aid executive function development in children 4 to 12 years old. Science.

[14] Best JR (2010). Effects of physical activity on children’s executive function: contributions of experimental research on aerobic exercise. Dev Rev.

[15] Howard SJ, Melhuish E (2016). An early years toolbox for assessing early executive function, language, self-regulation, and social development: Validity, reliability, and preliminary norms. J Psychoeduc Assess.

[16] Blair C, Razza RP (2007). Relating effortful control, executive function, and false belief understanding to emerging math and literacy ability in kindergarten. Child Dev.

[17] Schoemaker K, Mulder H, Deković M, Matthys W (2013). Executive functions in preschool children with externalizing behavior problems: a meta-analysis. J Abnorm Child Psychol.

[18] Smart D, Australian Institute of Family Studies (2011). How young children are faring: behaviour problems and competencies. The longitudinal study of Australian children annual statistical report 2010.

[19] Fergusson DM, John Horwood L, Ridder EM (2005). Show me the child at seven: the consequences of conduct problems in childhood for psychosocial functioning in adulthood. J Child Psych Psy.

[20] Griffiths LJ, Dowda M, Dezateux C, Pate R (2010). Associations between sport and screen-entertainment with mental health problems in 5-year-old children. Int J Behav Nutr Phys Act.

[21] Ebenegger V, Marques-Vidal P-M, Munsch S, Quartier V, Nydegger A, Barral J, Hartmann T, Dubnov-Raz G, Kriemler S, Puder JJ (2012). Relationship of hyperactivity/inattention with adiposity and lifestyle characteristics in preschool children. J Child Neurol.

[22] Fagot BI, O'Brien M. Activity level in young children: cross-age stability, situational influences, correlates with temperament, and the perception of problem behaviors. Merrill-Palmer Q. 1994;378–398.

[23] Slaughter V, Imuta K, Peterson CC, Henry JD (2015). Meta‐analysis of theory of mind and peer popularity in the preschool and early school years. Child Dev.

[24] Fink E, Begeer S, Hunt C, Rosnay M (2014). False-belief understanding and social preference over the first 2 years of school: a longitudinal study. Child Dev.

[25] Fink E, Begeer S, Peterson CC, Slaughter V, Rosnay M (2015). Friendlessness and theory of mind: a prospective longitudinal study. Br J Dev Psychol.

[26] Rosnay M, Hughes C (2006). Conversation and theory of mind: do children talk their way to socio‐cognitive understanding?. Br J Dev Psychol.

[27] Wellman HM, Liu D (2004). Scaling of theory‐of‐mind tasks. Child Dev.

[28] Devine RT, Hughes C (2014). Relations between false belief understanding and executive function in early childhood: a meta‐analysis. Child Dev.

[29] Nathanson AI, Sharp ML, Aladé F, Rasmussen EE, Christy K (2013). The relation between television exposure and theory of mind among preschoolers. J Commun.

[30] Wong TY, Shankar A, Klein R, Klein BE, Hubbard LD (2005). Retinal arteriolar narrowing, hypertension, and subsequent risk of diabetes mellitus. Arch Intern Med.

[31] Wong TY, Kamineni A, Klein R, Sharrett AR, Klein BE, Siscovick DS, Cushman M, Duncan BB (2006). Quantitative retinal venular caliber and risk of cardiovascular disease in older persons: the cardiovascular health study. Arch Intern Med.

[32] Wang JJ, Liew G, Klein R, Rochtchina E, Knudtson MD, Klein BE, Wong TY, Burlutsky G, Mitchell P (2007). Retinal vessel diameter and cardiovascular mortality: pooled data analysis from two older populations. Eur Heart J.

[33] Witt N, Wong TY, Hughes AD, Chaturvedi N, Klein BE, Evans R, McNamara M, Thom SAM, Klein R (2006). Abnormalities of retinal microvascular structure and risk of mortality from ischemic heart disease and stroke. Hypertension.

[34] Sasongko MB, Wong TY, Wang JJ (2010). Retinal arteriolar changes: intermediate pathways linking early life exposures to cardiovascular disease?. Microcirculation.

[35] Gopinath B, Wang J, Kifley A, Tan A, Wong T, Mitchell P (2013). Influence of blood pressure and body mass index on retinal vascular caliber in preschool-aged children. J Hum Hypertens.

[36] Gopinath B, Baur LA, Wang JJ, Hardy LL, Teber E, Kifley A, Wong TY, Mitchell P (2011). Influence of physical activity and screen time on the retinal microvasculature in young children. Arterioscler Thromb Vasc Biol.

[37] Department of Health. National Physical Activity Recommendations for Children (0–5 years). Australian Government. http://www.health.gov.au/internet/main/publishing.nsf/content/health-pubhlth-strateg-phys-act-guidelines#npa05. Accessed 21 Feb 2017.

[38] Tremblay MS, LeBlanc AG, Carson V, Choquette L, Connor Gorber S, Dillman C, Duggan M, Gordon MJ, Hicks A, Janssen I (2012). Canadian physical activity guidelines for the early years (aged 0–4 years). Appl Physiol Nutr Metab.

[39] Department of Health. Physical activity guidelines for Early Years (Under 5 s). United Kingdom Government. [https://www.gov.uk/government/uploads/system/uploads/attachment_data/file/213738/dh_128143.pdf. Accessed 21 Feb 2017.

[40] Skouteris H, Dell’Aquila D, Baur LA, Dwyer GM, McCabe MP, Ricciardelli LA, Fuller-Tyszkiewicz M (2012). Physical activity guidelines for preschoolers: a call for research to inform public health policy. Med J Aust.

[41] Australian Bureau of Statistics. Census of population and housing: Socio-economic indexes for areas (SEIFA). Data cube 2011. Australian Government; 2011. http://www.abs.gov.au/ausstats/abs@.nsf/DetailsPage/2033.0.55.0012011?OpenDocument. Accessed 21 Feb 2017.

[42] Pate RR, Almeida MJ, McIver KL, Pfeiffer KA, Dowda M (2006). Validation and calibration of an accelerometer in preschool children. Obesity.

[43] Cliff DP, Reilly JJ, Okely AD (2009). Methodological considerations in using accelerometers to assess habitual physical activity in children aged 0–5 years. J Sci Med Sport.

[44] Janssen X, Cliff DP, Reilly JJ, Hinkley T, Jones RA, Batterham M, Ekelund U, Brage S, Okely AD (2013). Predictive validity and classification accuracy of ActiGraph energy expenditure equations and cut-points in young children. PLoS ONE.

[45] Goodman R (2001). Psychometric properties of the strengths and difficulties questionnaire. J Am Acad Child Adolesc Psychiatry.

[46] Hawes DJ, Dadds MR (2004). Australian data and psychometric properties of the strengths and difficulties questionnaire. Aust NZ J Psychiatry.

[47] Pons F, Harris PL, de Rosnay M (2004). Emotion comprehension between 3 and 11 years: developmental periods and hierarchical organization. Eur J Dev Psychol.

[48] Schultz D, Izard CE, Bear G (2004). Children’s emotion processing: relations to emotionality and aggression. Dev Psychopathol.

[49] Slaughter V, de Rosnay M (2016). Theory of mind development in context.

[50] Eisenberg N, Shea CL, Carlo G, Knight GP (2014). Empathy-related responding and cognition: a “chicken and the egg” dilemma. Handbook of moral behavior and development: research.

[51] Hoffman ML. Empathy and moral development: Implications for caring and justice. Cambridge: Cambridge University Press; 2001.

[52] Bryant BK. An index of empathy for children and adolescents. Child Dev. 1982;413–425.

[53] Santucci AK, Silk JS, Shaw DS, Gentzler A, Fox NA, Kovacs M (2008). Vagal tone and temperament as predictors of emotion regulation strategies in young children. Dev Psychobiol.

[54] Balle M, Bornas X, Tortella-Feliu M, Llabrés J, Morillas-Romero A, Aguayo-Siquier B, Gelabert JM (2013). Resting parietal EEG asymmetry and cardiac vagal tone predict attentional control. Biol Psychol.

[55] Gutin B, Owens S, Slavens G, Riggs S, Treiber F (1997). Effect of physical training on heart-period variability in obese children. J Pediatr.

[56] Jennings JR, Kamarck T, Stewart C, Eddy M, Johnson P (1992). Alternate cardiovascular baseline assessment techniques: vanilla or resting baseline. Psychophysiology.

[57] Knudtson MD, Lee KE, Hubbard LD, Wong TY, Klein R, Klein BE (2003). Revised formulas for summarizing retinal vessel diameters. Curr Eye Res.

[58] Cheung CYL, Hsu W, Lee ML, Wang JJ, Mitchell P, Lau QP, Hamzah H, Ho M, Wong TY (2010). A new method to measure peripheral retinal vascular caliber over an extended area. Microcirculation.

[59] Sanson A, Nicholson J, Ungerer J, Zubrick S, Wilson K, Ainley J, Berthelsen D, Broom D, Harrison L, Rodgers B (2002). Introducing the longitudinal study of Australian children-LSAC discussion paper no. 1.

[60] Sammons P, Toth K, Sylva K, Melhuish E, Siraj I, Taggart B (2015). The long-term role of the home learning environment in shaping students’ academic attainment in secondary school. J Child Serv.

